# MRI Assessment of Energy Loss Within the Thoracic Aorta and Its Impact on Cardiac Function in Fontan Patients After Aortic Reconstruction

**DOI:** 10.1002/jmri.70025

**Published:** 2025-06-30

**Authors:** Yujiro Ide, Dominik Gabbert, Jan Hinnerk Hansen, Anselm Uebing, Inga Voges

**Affiliations:** ^1^ Department of Congenital Heart Disease and Pediatric Cardiology University Hospital Schleswig‐Holstein Campus Kiel Germany; ^2^ German Center for Cardiovascular Research (DZHK) Partner Site Hamburg/Greifswald/Kiel/Lübeck Kiel Germany

**Keywords:** 4D flow, congenital heart disease, Fontan circulation

## Abstract

**Background:**

In Fontan patients undergoing aortic reconstruction, concerns regarding the impact of aortic function on ventricular function exist.

**Purpose:**

4D Flow MRI was used to compare energy loss (EL) within the thoracic aorta in patients with and without aortic reconstruction.

**Study Type:**

Retrospective case control.

**Population:**

Eighty‐nine patients underwent 4D Flow MRI: group A (*n* = 36), Fontan patients without aortic reconstruction (9.9 (1.0–26.7) years since Fontan completion); group B (*n* = 42), Fontan patients with aortic reconstruction (11.8 (1.0–26.4) years since Fontan completion); and group C (*n* = 11), patients with biventricular circulation without aortic reconstruction.

**Field Strength/Sequence:**

Balanced SSFP cine and time‐resolved 3D phase contrast (4D Flow) sequences at 1.5 T or 3 T.

**Assessment:**

Peak and average aortic EL in the thoracic aorta as well as peak aortic velocity and flow volume were assessed. Correlations between EL indexed to aortic forward flow volume and volumetric ventricular parameters and peak aortic velocity were assessed.

**Statistical Tests:**

Kruskal‐Wallis test, chi‐square test and Spearman's correlation coefficient were used.

**Results:**

Peak and average EL were significantly larger in group B than in groups A and C (peak EL (mW); A: 1.45 (0.22–9.81), B: 3.09 (0.51–12.49), C: 2.10 (1.20–3.45); average EL (mW); A: 0.46 (0.07–2.63), B: 1.13 (0.13–4.67), C: 0.76 (0.40–1.98)). Group B had significantly larger ventricular end‐diastolic volume index (EDVi, 108 mL/m^2^) and end‐systolic volume index (ESVi, 53 mL/m^2^), significantly lower ejection fraction (EF, 51%) and significantly greater end‐diastolic myocardial mass (MM, 50 g/m^2^) of the systemic ventricle than group A (EDVi: 86 mL/m^2^, ESVi: 34 mL/m^2^, EF: 58%, end‐diastolic MM: 43 g/m^2^). In Fontan patients, indexed average aortic EL correlated positively with aortic peak velocity (*R* = 0.68) and with years after Fontan completion (*R* = 0.60).

**Data Conclusion:**

Fontan patients who underwent aortic reconstruction had increased aortic EL, even in the absence of significant residual aortic stenosis.

**Evidence Level:**

Level 3.

**Technical Efficacy:**

Stage 3.

**Summary**

- Plain language summary
  ○ This study used 4D flow MRI to measure energy loss (EL) in the thoracic aorta of patients with a Fontan circulation, both with and without aortic reconstruction.
  ○ These patients were compared with patients who had a biventricular circulation and had not undergone aortic reconstruction.
  ○ Peak and average EL in the thoracic aorta were significantly higher in Fontan patients who had undergone aortic reconstruction than in those patients who had not.
  ○ EL indexed to aortic forward flow volume correlated with aortic peak velocity and the years since Fontan completion.
  ○ Following aortic reconstruction, EL may serve as a useful hemodynamic parameter.

## 1 Introduction

Aortic arch obstruction or hypoplasia is a common finding in patients with complex congenital heart disease undergoing the Fontan pathway, particularly in those with hypoplastic left heart syndrome (HLHS) [1]. These patients require extensive aortic reconstruction that involves the ascending aorta, the aortic arch, and the aortic isthmus [2]. Since the introduction of aortic reconstruction surgeries such as the Norwood [3] and the Damus–Kaye–Stansel (DKS) procedures [4, 5, 6], surgical techniques have become more sophisticated, and developments in perioperative care have improved patient outcomes, making Fontan completion possible for many of them today [7]. However, residual stenosis in the reconstructed aorta is a common complication and may necessitate surgical or catheter‐based re‐interventions [8, 9]. In addition, several studies have shown that the function of the reconstructed aorta can impact ventricular function [10, 11].

With the development of newer imaging methods, a more detailed morphological and functional aortic assessment has become possible. Recent studies have shown that 4‐dimensional flow (4D flow) MRI enables not only the evaluation of aortic morphology and blood flow, but also the measurement of advanced hemodynamic markers such as fluid energy loss (EL), which is the dissipated energy with blood viscosity during the cardiac cycle [12, 13]. EL allows assessment of cardiac function and hemodynamics in a wide range of cardiovascular diseases, including valvular heart disease and aortopathies as well as congenital heart diseases [14, 15]. It has also been reported that EL measurements can be used to refine the techniques of surgical aortic reconstruction [16].

The hypothesis of this study was that changes in aortic EL may represent changes in cardiac workload that cannot be detected by conventional methods based on vascular morphology and pressure changes, and that EL may be a useful additional hemodynamic parameter, particularly for Fontan patients without an apparent residual problem after aortic reconstruction. Thus, the aim of this study was to compare thoracic aortic EL in Fontan patients with and without aortic arch reconstruction and in patients with biventricular circulation using 4D flow MRI.

## 2 Materials and Methods

### 2.1 Ethics Statement

Approval for this study was obtained from the local ethics committee of (No. D588/24). Parents, guardians, or patients signed a research consent at the time of the MRI examination.

### 2.2 Patients

This study was a retrospective review of MR images obtained between January 2023 and December 2023. All examinations were performed for routine surveillance. Patients with a functional single ventricle after Fontan completion with and without aortic arch reconstruction and patients with biventricular circulation who did not undergo reconstructive aortic surgery were included. Another inclusion criterion was the availability of a complete 4D flow data set. Patients were excluded if they had residual stenosis (peak velocity > 2.5 m/s in 4D flow sequence) in the ascending aorta, aortic arch, or descending aorta, or if an aortic stent had been placed.

Eighty‐nine patients met the inclusion criteria and they were allocated to the following groups:

- Group A (*n* = 36): Fontan patients without surgical aortic reconstruction
- Group B (*n* = 42): Fontan patients who had reconstructive aortic surgery (Norwood or DKS operation)
- Group C (*n* = 11): patients with biventricular circulation without aortic surgery.

Among group A group B, and group C, there were no significant differences in age (*p* = 0.83) and body surface area (*p* = 0.10). In group A, 26 (72%) patients had a systemic left ventricle, and eight (22%) had a systemic right ventricle. In group B, six (14%) patients had a systemic left ventricle, and 36 (86%) had a systemic right ventricle. In group C, all 11 patients (4, post‐repair for atrial septal defect (ASD) or ventricular septal defect (VSD); 3, myocarditis; 2, cardiomyopathy; 2, arrhythmias) had normal biventricular circulation. The detailed patient characteristics, including cardiac diagnosis and dominant systemic ventricle, are shown in Table 1.

**TABLE 1:** Patient characteristics.

	Group A	Group B	Group C	p*
*N*	36	42	11	
Age (years)	13.7 (4.10–40.30)	14.7 (3.90–31.20)	16.8 (4.50–19.30)	0.832
BSA (m^2^)	1.36 (0.63–2.25)	1.48 (0.64–2.20)	1.69 (0.79–2.28)	0.098
Sex (F/M)	21/15	14/28	9/2	**0.006**
Years since Fontan completion	9.9 (0.99–26.70)	11.8 (0.99–26.40)	—	0.474
Type of Fontan operation (lateral tunnel/extracardiac conduit)	23/13	35/7	—	0.069
Cardiac diagnosis (N)				< **0.001**
Systemic left ventricle	TA: 13	TA: 1	ASD or VSD: 4	
	DILV: 6	DILV: 4	s/p myocarditis: 3	
	PAIVS: 5	IAA + hypoplastic RV: 1	cardiomyopathy: 2	
	uAVSD: 2		arrhythmia: 2	
Systemic right ventricle	DORV: 6	HLHS: 34	—	
	ccTGA: 2	cAS: 2	—	
Ambiguous systemic ventricle	2	—	—	

Abbreviations: ASD, atrial septal defect; BSA, body surface area; cAS, critical aortic stenosis; ccTGA, congenitally corrected transposition of the great arteries; DILV, double inlet left ventricle; HLHS, hypoplastic left heart syndrome; IAA, interruption of the aortic arch; PAIVS, pulmonary atresia with intact ventricular septum; RV, right ventricle; s/p, status post; TA, tricuspid atresia; uAVSD, unbalanced atrioventricular septal defect; VSD, ventricular septal defect. *p‐values shown in bold are statistically significant with the significance level set at *p* < 0.05.

### 2.3 MRI Acquisition

MR images were obtained with 1.5 Tesla (T) (MAGNETOM Aera, Siemens Healthcare, Erlangen, Germany) or 3T (Ingenia, Philips Healthcare, Best, the Netherlands) scanners. ECG‐gated steady‐state free precession cine images were acquired to measure ventricular function, mass, and volume. The scan parameters were: field of view 175–450 mm, slice thickness 5–8 mm, 20–30 cardiac phases, no slice gap. To measure blood flow and EL within the thoracic aorta, a 4D flow sequence was used. Scan parameters were as follows: echo time = 2.2–3.1 ms, repetition time = 5.0–5.6 ms, flip angle = 5°–7°, velocity encoding = 110–350 cm/s, field of view 300–360 × 220–280 mm^2^, voxel size = 2.4 × 2.4 × 2.5 mm^3^, temporal resolution on average = 24 ms. In adolescent and adult patients, the images were acquired during breath‐holding (on average 15 cardiac cycles). In young and uncooperative patients, MRI was performed under sedation with midazolam as premedication (dosage: 0.1 mg/kg) and propofol (bolus doses of propofol with a dosage of 0.5 mg/kg were followed by continuous infusion of propofol with a dosage of 2–3 mL/kg/h) during free breathing and respiratory navigator‐gating for the 4D flow sequence.

### 2.4 MRI Postprocessing

**FIGURE 1:**
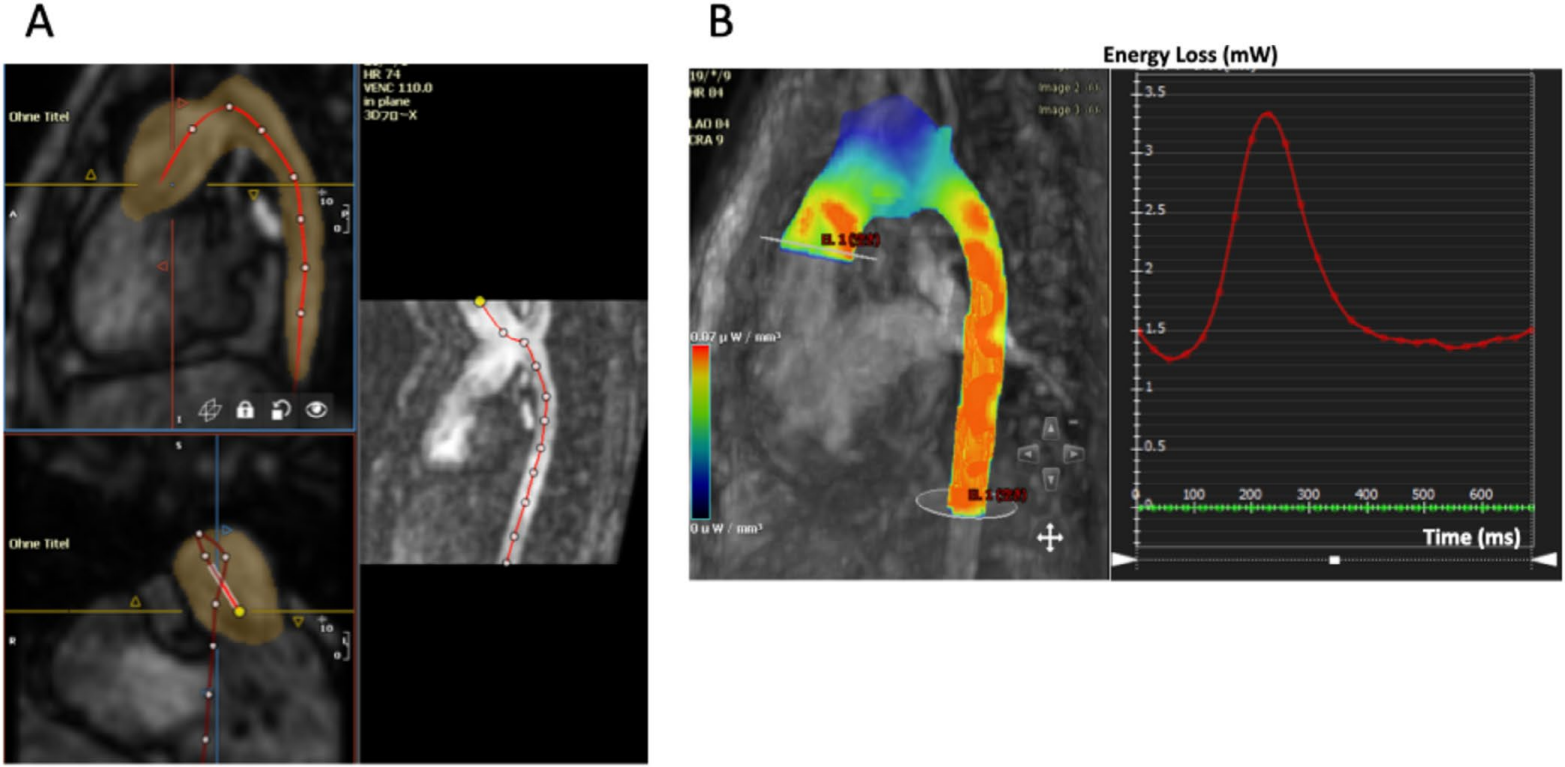
(a) Following segmentation of the thoracic aorta, a center line from the aortic root to the descending aorta at the level of the diaphragm is manually drawn. The thoracic aorta is presented in sagittal view (top left) and frontal view (bottom left). A curved multiplanar reconstruction image (right) is automatically generated. (b) From the aortic root to the descending aorta at the level of the diaphragm, within the same region as in (a), both peak and average energy loss in one cardiac cycle are calculated.

MR images were analyzed with Cvi42 (Circle, Cardiovascular Imaging, Calgary, Canada) in cooperation between 2 observers with 1 year (YI) and 18 years (IV) of experience. First3 background phase offset correction was performed and then semi‐automatic aortic segmentation from 4D flow data sets using the 3D region growing method was carried out. Following this, a center line from the neo‐aortic valve or the original aortic valve to the diaphragm level was manually drawn (Figure 1a). Total forward flow (mL/cardiac cycle) in the ascending aorta was measured at the level of the sinotubular junction perpendicular to the center line. In addition, a perpendicular plane was manually placed at the highest aortic flow velocity to measure the peak velocity. Within the same region of the thoracic aorta, peak EL (pEL) and average EL (aEL) were calculated automatically (Figure 1b).

EL is generated by friction between blood and the vessel wall and by turbulence in blood flow, and increases in areas where the flow collides and where the flow separates and rubs against itself [17]. The formula for calculating EL varies slightly among software packages, but the essential component is the velocity vector component in the Cartesian coordinate system, which is measured non‐invasively using MRI [17, 18].

From cine images, the degree of atrioventricular valve regurgitation (AVVR) was visually determined by 2 observers and classified as none = 0, trivial = 1, mild = 2, moderate = 3, or severe = 4. Volumetric parameters, including end‐diastolic volume (EDV), end‐systolic volume (ESV), stroke volume (SV), end‐diastolic myocardial mass (EDMM), and ejection fraction (EF) of the systemic ventricle were measured semi‐automatically from short‐axis cine images [19]. Measured ventricular volumes and myocardial mass were indexed to body surface area (EDVi, ESVi, SVi, EDMMi) determined using the Mosteller formula [20].

EL and cardiac parameters were compared among the three groups (groups A–C). Furthermore, in Fontan patients (groups A and B), the correlations between EL and each cardiac parameter including degree of AVVR, years elapsed since Fontan completion, and peak velocity of blood flow in the thoracic aorta were examined.

### 2.5 Statistical Analysis

All statistical analyses were performed with EZR (Saitama Medical Center, Jichi Medical University, Saitama, Japan), a graphical user interface for R (The R Foundation for Statistical Computing, Vienna, Austria). More precisely, it is a modified version of R commander designed to add statistical functions frequently used in biostatistics [21].

Continuous variables were presented as median with range for non‐normally distributed data after investigating normality using the Shapiro‐Wilk test. The variables among the three groups were compared using ANOVA for normally distributed data or the Kruskal‐Wallis test for non‐normally distributed data. The chi‐square test was used to compare categorical variables. In the case of multiple pairwise comparisons, the level of significance was adjusted by Bonferroni correction. Correlations were assessed using univariable regression analysis with nonparametric Spearman's r value. We conducted a multiple regression analysis with a forward–backward stepwise selection method using Akaike's information criterion to identify variables involved in EL. Statistical significance was set at *p* < 0.05.

## 3 Results

### 3.1 Aortic Flow Parameters and Ventricular Function

Total forward flow in the ascending aorta was significantly larger in group C (77 (27–128) mL) than in the other two groups, but there was no difference between group A (48 (20–122) mL) and group B (53 (21–112) mL) (*p* = 1.0). Peak velocity of aortic blood flow was significantly lower in group A (1.15 (0.66–2.45) m/s) than in the other two groups (group B: 1.62 (0.71–2.50) m/s, group C: 1.47 (1.16–1.72) m/s). The pEL (3.09 (0.51–12.49) mW) and aEL (1.13 (0.13–4.67) mW), as well as the indexed values using flow volume at the ascending aorta, were significantly higher in group B than in the other two groups. Furthermore, patients in group B had a significantly larger EDVi (108 (43–262) mL/m^2^) and ESVi (53 (16–209) mL/m^2^), lower EF (51% (20%–63%)) and greater EDMMi (50 (20–80) g/m^2^) than patients in group A. Detailed results are shown in Tables 2 and 3.

**TABLE 2:** MRI analysis.

	Group A (*n* = 36)	Group B (*n* = 42)	Group C (*n* = 11)	* *p*
Systemic ventricular status				
EDVi (mL/m^2^)	86 (48–137)	108 (43–263)	92 (71–117)	< 0.001
ESVi (mL/m^2^)	34 (16–65)	53 (16–210)	36 (22–50)	< 0.001
EF (%)	58 (41–69)	51 (20–63)	57 (50–78)	< 0.001
EDMMi (g/m^2^)	43 (21–70)	50 (22–80)	49 (39–97)	0.047
Grade of AVVR	0.33 ± 0.59	1.02 ± 084	0.00	< 0.001
Flow analysis				
Flow volume at AAo (mL/cycle)	48 (20–122)	53 (21–112)	77 (27–128)	0.024
Peak velocity within the thoracic aorta (m/s)	1.15 (0.66–2.45)	1.62 (0.71–2.50)	1.47 (1.16–1.72)	< 0.001
aEL (mW)	0.46 (0.07–2.63)	1.13 (0.13–4.67)	0.76 (0.40–1.98)	0.011
pEL (mW)	1.45 (0.22–9.81)	3.09 (0.51–12.49)	2.10 (1.20–3.45)	0.031
aEL/BSA (mW/m^2^)	0.33 (0.08–1.44)	0.67 (0.14–2.13)	0.49 (0.29–1.05)	0.003
pEL/BSA (mW/m^2^)	1.22 (0.26–4.36)	1.99 (0.47–8.33)	1.30 (0.76–2.28)	0.004
Indexed aEL (mW/mL/cycle)	0.01 (0.00–0.03)	0.02 (0.00–0.06)	0.01 (0.01–0.02)	0.003
Indexed pEL (mW/mL/cycle)	0.03 (0.01–0.12)	0.05 (0.01–0.26)	0.03 (0.02–0.07)	0.001

Abbreviations: AAo, ascending aorta; aEL, average energy loss; AVVR, atrioventricular valve regurgitation; BSA, body surface area; EDMMi, indexed end‐diastolic myocardial mass; EDVi, indexed end‐diastolic volume; EF, ejection fraction; ESVi, indexed end‐systolic volume; pEL, peak energy loss.

* *p* value is obtained from the Kruskal‐Wallis test, which was conducted to compare three groups.

**TABLE 3:** Post hoc test.

	Group A vs. B	Group A vs. C	Group B vs. C
Systemic ventricular status			
EDVi (mL/m^2^)	< **0.001**	0.600	0.173
ESVi (mL/m^2^)	< **0.001**	1.00	**0.017**
EF (%)	< **0.001**	1.00	**0.0071**
EDMMi (g/m^2^)	0.14	0.18	1.00
Flow analysis			
Flow volume at AAo (mL/cycle)	1.00	0.053	**0.022**
Peak velocity within the thoracic aorta (m/s)	< **0.001**	**0.0025**	0.36
aEL (mW)	**0.012**	0.25	1.00
pEL (mW)	**0.032**	0.84	0.97
aEL/BSA (mW/m^2^)	**0.0024**	0.39	0.94
pEL/BSA (mW/m^2^)	**0.0091**	1.00	**0.041**
Indexed aEL (mW/mL/cycle)	**0.0033**	1.00	0.36
Indexed pEL (mW/mL/cycle)	**0.0046**	1.00	**0.0064**

Abbreviations: AAo, ascending aorta; aEL, average energy loss; AVVR, atrioventricular valve regurgitation; BSA, body surface area; EDMMi, indexed end‐diastolic myocardial mass; EDVi, indexed end‐diastolic volume; EF, ejection fraction; ESVi, indexed end‐systolic volume; pEL, peak energy loss.

*p* values were obtained after Bonferroni correction. Those *p* values shown in bold are statistically significant with the signicance level set at *p* < 0.05.

### 3.2 Correlation Between EL and Cardiac Parameters as Well as Clinical Data in Patients With Fontan Circulation

**FIGURE 2:**
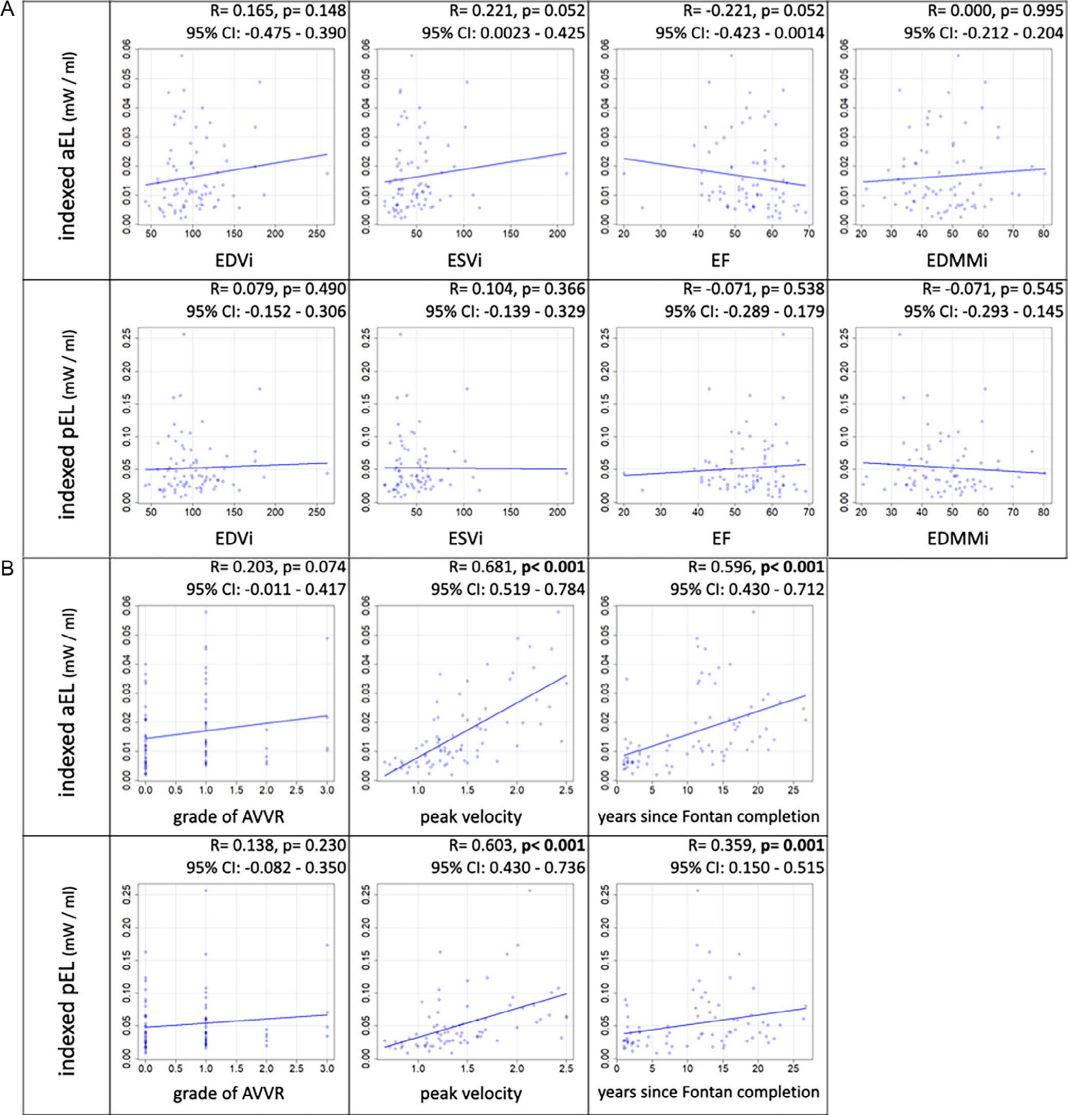
Correlation between indexed average and peak EL with (a) EDVi, ESVi, EF, EDMMi, and (b) grade of AVVR, peak velocity within the thoracic aorta, and years after Fontan completion in Fontan patients (group A + group B, *n* = 78). AVVR, atrioventricular valve regurgitation; CI, confidence interval; EDMMi, indexed end‐diastolic myocardial mass; EDVi, indexed end‐diastolic volume; EF, ejection fraction; EL, energy loss; ESVi, indexed end‐systolic volume.

Both indexed aEL and pEL had significant positive correlations with peak velocity within the thoracic aorta (aEL: *R* = 0.681, 95% CI 0.519–0.784; pEL: *R* = 0.603, 95% CI 0.430–0.736) and with years after Fontan completion (aEL: *R* = 0.596; 95% CI 0.428–0.712; pEL: *R* = 0.359, 95% CI; 0.150–0.515); however, neither of them had a significant correlation with EDVi (aEL: *p* = 0.148; pEL: *p* = 0.490), EDMMi (aEL: *p* = 0.995; pEL: *p* = 0.545), and the degree of AVVR (aEL: *p* = 0.074; pEL: *p* = 0.230) (Figure 2a,b). Correlations with peak velocity and years after Fontan completion were more pronounced for indexed aEL (*R* = 0.681 and 0.596, respectively) than for pEL (*R* = 0.603 and 0.359, respectively) (Figure 2b). There were trends of correlation between the indexed aEL and ESVi (*R* = 0.22, *p* = 0.052) as well as EF (*R* = −0.22, *p* = 0.052).

### 3.3 EL and the Presence or Absence of Aortic Arch Reconstruction

**FIGURE 3:**
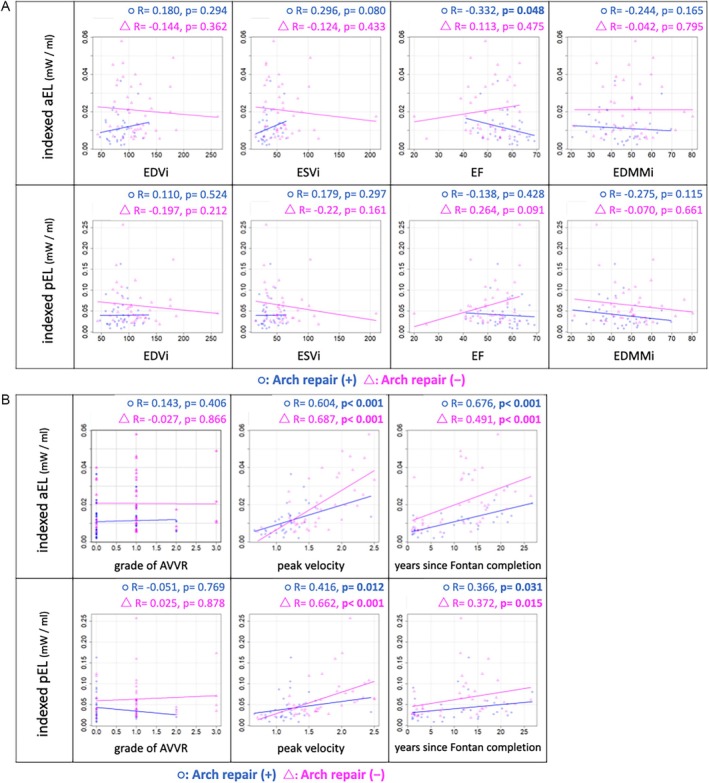
Comparison of correlation between indexed average and peak EL with (a) EDVi, ESVi, EF, EDMMi, and (b) grade of AVVR, peak velocity within the thoracic aorta, and years after Fontan completion in patients with aortic arch repair (group B, *n* = 42, pink) and those without (group A, *n* = 36, blue). AVVR, atrioventricular valve regurgitation; EDMMi, indexed end‐diastolic myocardial mass; EDVi, indexed end‐diastolic volume; EF, ejection fraction; EL, energy loss; ESVi, indexed end‐systolic volume.

A borderline significant negative correlation was observed between EF and aEL only in patients without aortic arch reconstruction (*R* = ‐0.332, *p* = 0.048). Apart from this, no significant correlations were found between aEL and EDVi (*p* = 0.294, 0.362), ESVi (*p* = 0.080, 0.433) dimensions, EF, and EDMMi (*p* = 0.165, 0.795), regardless of the aortic arch reconstruction status (Figure 3a). A significant positive correlation was found between peak velocity and both aEL and pEL in both groups A and B. Additionally, a moderate correlation was observed between the years after Fontan completion and aEL (Figure 3b; with aortic arch repair: *R* = 0.491; without aortic arch repair: *R* = 0.676), with a significantly larger aEL in patients who underwent aortic arch repair compared to those who did not (Table 4).

**TABLE 4:** Correlation between indexed average EL or indexed peak EL and clinical parameters.

		Arch repair (−)			Arch repair (+)	
	*R*	95% CI	*p*	*R*	95% CI	*p*
Indexed aEL						
EDVi (mL/m^2^)	0.180	−0.148–0.477	0.294	−0.144	−0.441–0.156	0.362
ESVi (mL/m^2^)	0.296	−0.025–0.552	0.080	−0.124	−0.439–0.191	0.433
EF (%)	−0.332	−0.564–0.031	**0.048**	0.113	−0.199–0.390	0.475
EDMMi (g/m^2^)	−0.244	−0.528–0.089	0.165	−0.042	−0.349–0.276	0.795
Grade of AVVR	0.143	−0.149–0.424	0.406	−0.027	−0.327–0.289	0.866
Peak velocity within the thoracic aorta (m/s)	0.604	0.319–0.769	< **0.001**	0.687	0.492–0.827	< **0.001**
Years after Fontan completion	0.676	0.445–0.821	< **0.001**	0.491	0.209–0.699	< **0.001**
Indexed pEL						
EDVi (mL/m^2^)	0.110	−0.229–0.434	0.524	−0.197	−0.516–0.125	0.212
ESVi (mL/m^2^)	0.179	−0.156–0.446	0.297	−0.220	−0.512–0.132	0.161
EF (%)	−0.138	−0.434–0.191	0.428	0.264	−0.065–0.571	0.091
EDMMi (g/m^2^)	−0.275	−0.541–0.0015	0.115	−0.070	−0.414–0.250	0.661
Grade of AVVR	−0.051	−0.345–0.233	0.769	0.025	−0.269–0.024	0.878
Peak velocity within the thoracic aorta (m/s)	0.416	0.127–0.655	**0.0012**	0.662	0463–0.793	< **0.001**
Years after Fontan completion	0.366	0.053–0.630	**0.0031**	0.372	0.070–0.605	**0.015**

Abbreviations: aEL, average energy loss; AVVR, atrioventricular valve regurgitation; CI, confident interval; EDMMi, indexed end‐diastolic myocardial mass; EDVi, indexed end‐diastolic volume; EF, ejection fraction; ESVi, indexed end‐systolic volume; pEL, peak energy loss.

### 3.4 Factors Affecting Average EL in Patients With a Fontan Circulation

By multiple linear regression, peak velocity within the thoracic aorta and years since Fontan completion were identified to have significant associations with indexed aEL (Table 5).

**TABLE 5:** Results of multiple regression analysis.

Variables	Estimate	Standardized	Std. error	*t* value	p*
(Intercept)	−0.0180	NA	0.0184	−0.977	0.332
Grade of AVVR	0.00186	0.119	0.0015	1.214	0.229
EDVi	−0.00010	−0.289	0.00014	−0.727	0.470
EF	0.00019	0.131	0.00026	0.638	0.525
ESVi	0.00013	0.291	0.00021	0.623	0.535
EDMMi	−0.00005	−0.044	0.00014	−0.345	0.731
Peak velocity within the thoracic aorta	0.0165	0.616	0.0025	6.687	< **0.001**
Years since Fontan completion	0.00050	0.291	0.00016	3.090	**0.00293**

Abbreviations: AVVR, atrioventricular valve regurgitation; EDMMi, indexed end‐diastolic myocardial mass; EDVi, indexed end‐diastolic volume; EF, ejection fraction; ESVi, indexed end‐systolic volume; Std. error, standard error. *P‐values shown in bold are statistically significant with the significance level set at *p* < 0.05.

### 3.5 Differences in EL due to Variations in Aortic Geometry

**FIGURE 4:**
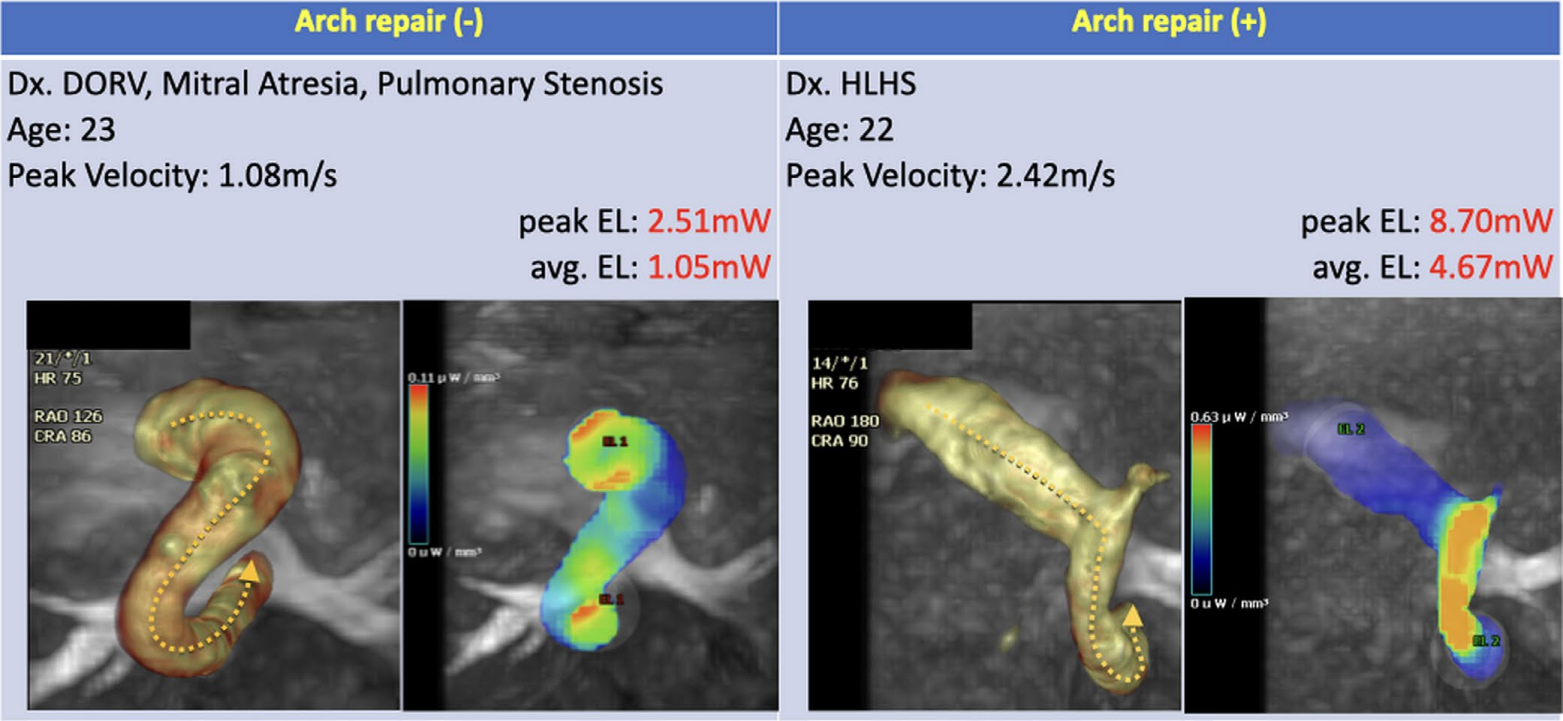
Three‐dimensional images of the reconstructed aorta in an example patient with large average EL (4.67 mW, right) and in an example patient with small average EL (1.05 mW, left). When viewed from the cranial perspective, the aorta with larger EL has a polyline shape, whereas the aorta with lower EL has a smooth inverted S‐shaped curve.

As comparative geometric examples, three‐dimensional images of the aorta, which represent both large aEL (4.67 mW) and small aEL (1.05 mW), are shown in Figure 4.

## 4 Discussion

This study showed that Fontan patients who underwent aortic reconstruction have larger EL within the thoracic aorta compared to those who did not undergo aortic reconstruction, despite having no apparent residual stenosis. Furthermore, EL increased over time after the completion of the Fontan procedure.

EL is a biomechanical parameter that measures how much energy the aorta absorbs during the cardiac cycle [22, 23]. Qiao et al. have proposed that in a healthy aorta, EL is composed of viscous friction, turbulent dissipation, and wall deformation [22, 23]. They have also stated that the proportion of EL due to turbulent dissipation depends on the geometry of the aorta. It can therefore be speculated that patients whose aortic geometry differs from normal would have a larger EL. Moreover, the results of the current study suggest that there was a higher EL within the aorta in Fontan patients who underwent aortic arch reconstruction, even in the absence of a significant stenosis, compared to the normal aorta.

EL is larger in patients with significant stenosis, as Fujisue et al. reported using computational fluid dynamics [24]. In clinical practice, a pressure gradient of 20 mmHg or more, measured invasively, is often used as a criterion for reintervention in cases of aortic arch obstruction [8, 9, 25]. It is noteworthy that in the current study, there was a significant positive correlation between EL and peak velocity within the aorta, even though patients with significant flow acceleration in the aorta were excluded. Oka et al. have reported a disturbed pattern of aortic blood flow and an increase in peak EL in a patient after coarctation repair, despite the absence of a significant pressure gradient [26]. Furthermore, Miyazaki et al. have reported a significant decrease in EL and the preservation of ventricular function after reoperation in patients with increased EL but no residual stenosis in the aortic arch after the Norwood operation [16]. The relationship between the degree of pressure gradient and the absolute value of EL is unclear, but EL may be a parameter that describes cardiac workload more precisely.

There are several possible reasons for the increase in EL with age after Fontan completion. The first is the dilation of the aorta. Aortic dilatation in patients who undergo the Norwood operation or the DKS procedure is common [27, 28]. In one study, it was reported that 98% of post‐Fontan patients had an aortic root z‐score of > 2 at a median follow‐up of 9.2 years [27]. Furthermore, Leiva et al. have reported that the neo‐aortic root and aortic arch are enlarged shortly after aortic arch reconstruction and continue to enlarge disproportionately until 12 months of age, followed by a gradual decline in enlargement through adolescence. Even after patients reach adolescence, enlargement with a *Z* value of 3 or greater was observed in the neo‐aortic root, ascending aorta, and transverse arch [28]. Barker et al. reported that EL was significantly elevated in the thoracic aorta of patients with a dilated aorta [18]. This association between aortic dilation and elevated EL was also found in patients who underwent the Norwood operation [29].

The second possible reason for increasing EL with years after Fontan completion is the increase in aortic wall stiffness. It is known that aortic stiffness increases with age due to histological changes in the aortic wall [30, 31]. Chung et al. has reported histological changes (fragmentation of elastin, loss of smooth muscle cells, and replacement with collagen) in the aorta with an increased EL, which was calculated from the stress–strain relationship curve, and a correlation between EL and the collagen/elastin ratio [15]. Therefore, it is thought that EL in the aorta increases with age. In patients after the Norwood procedure, a decreased distensibility of the ascending aorta and the aortic arch compared to healthy controls has been demonstrated [10], suggesting an increased aortic stiffness, which is expected to promote an increase in EL.

For these reasons, it can be speculated that EL in the thoracic aorta increases gradually, even after the Fontan procedure. However, longitudinal data follow‐up over time in the same patients would be needed to clarify this.

Even if EL is currently small in the studied Fontan cohort, larger EL in the future might increase cardiac workload, which could contribute to ventricular dysfunction. For this reason, regular follow‐up with MRI according to consensus agreements may be clinically useful [32].

Although the aortic geometry was not examined in detail in this study, there seems to be a characteristic shape in a large EL aorta. As representative geometric examples, Example three‐dimensional images of large aEL and small aEL aortae have been presented. When viewed from the cranial side, a high EL aorta is characterized by a polyline shape of the ascending aorta and aortic arch, rather than a smooth inverted S‐shaped curve, which is seen in a small EL aorta. Presumably, the shape of the reconstructed aorta, which requires a steep change in the direction of blood flow, leads to increased EL. Future improvements in surgical techniques are expected so that aortic reconstruction is performed not only to avoid the occurrence of stenosis but also to achieve less EL. For this, it may be helpful to utilize patient‐specific three‐dimensional aortic patches based on the information obtained from preoperative imaging, as proposed by Belitsis et al. [33].

## 5 Limitations

The study was limited by the use of different vendors, which may have impacted the results, as well as by the small number of patients with various types of congenital heart disease, and by the retrospective nature of the study design. Few patients required sedation for the MRI scan, which could have affected vascular tone and study results. The study results may also have been influenced by the status of the fenestration in patients with a Fontan circulation. Longitudinal changes in EL within the same patients were not measured because 4D flow for assessing aortic blow flow was introduced in our department only a few years ago. Furthermore, the relationship between EL values and severity of disease was not investigated in this study.

## 6 Conclusions

Fontan patients who underwent aortic reconstructive surgery have increased aortic EL, even in the absence of significant residual stenosis. Furthermore, an increase in EL may be associated with an increase in end‐systolic volume and a decrease in ejection fraction. Additionally, since EL gradually rises even after Fontan completion, regularly measuring EL may have potential to serve as a useful hemodynamic parameter for long‐term follow‐up of patients after aortic reconstruction.

## Conflicts of Interest

The authors declare no conflicts of interest.
